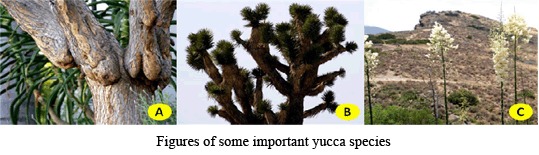# Yucca: A medicinally significant genus with manifold therapeutic attributes

**DOI:** 10.1007/s13659-012-0090-4

**Published:** 2012-12-03

**Authors:** Seema Patel

**Affiliations:** Better Process Control School, Department of Food Science and Technology, University of California Davis, California, USA

**Keywords:** Yucca, saponin, antioxidant, cytotoxicity, antimicrobial

## Abstract

The genus Yucca comprising of several species is dominant across the chaparrals, canyons and deserts of American South West and Mexico. This genus has long been a source of sustenance and drugs for the Native Americans. In the wake of revived interest in drug discovery from plant sources, this genus has been investigated and startling nutritive and therapeutic capacities have come forth. Apart from the functional food potential, antioxidant, antiinflammation, antiarthritic, anticancer, antidiabetic, antimicrobial, and hypocholesterolaemic properties have also been revealed. Steroidal saponins, resveratrol and yuccaols have been identified to be the active principles with myriad biological actions. To stimulate further research on this genus of multiple food and pharmaceutical uses, this updated review has been prepared with references extracted from MEDLINE database.

### Introduction

The genus Yucca belongs to Asparagaceae family and encompasses about 40–50 medicinally potent plants. These flowering plants generally thrive in arid parts of Southwestern US and Mexico, namely Mojave, Sonoran, Colorado and Chihuahuan Deserts, chaparrals of coastal California, sand dunes and Baja California. However, the species *Yucca alofolia* (Spanish bayonet) grows in Southeastern USA. *Yucca whipplei* (Chaparral yucca) is prevalent in the south California chaparral. *Yucca brevifolia* (Joshua tree) is the signature plant of Mojave Desert. *Yucca schidigera* (Mojave yucca) is prevalent across Mojave and Sonoran Deserts and regarded highly for its pharmaceutical values. *Yucca elata* (Soaptree yucca) is another distinct species in Sonoran and Chihuahua Deserts. *Yucca elephantipes* (Giant yucca) is often planted for landscape purpose in urban area. Figures of some important yucca species have been presented as Fig. 1.

Apart from being a source of wide range of utilitarian products, this genus has found reputed place in folk medicine. These indigenous flowering succulent plants held ethno-botanical importance for Native Americans. The plant extracts were used to soothe joint pain, bleeding, urethral and prostate inflammations. They used the brewed leaves for common ailments as psoriasis, dandruff, hair loss and skin sores. Navajo Indians used it on sunburns and scratches. In northern New Mexico, healers used a tea from the leaves and roots to treat asthma and headache. The Catawba, Cherokee, Nanticoke and other Native American tribes used *Yucca filamentosa* as medicines and soap. The roots were crushed to make poultice for wound healing. Further, the roots were used to cure gonorrhoea and rheumatism. The Zuni tribe inhabiting the western New Mexico region used *Yucca elata* sap as hair growth stimulant. The infusion of the crushed leaves of *Y. baccata* has been used as an antiemetic and the fruits have been eaten raw as a laxative. Skin emollient, soporific agents and antidiabetic are other reported uses. The Chumash Indians consumed root, leaves and flower stalk.

Now, this genus is receiving scientific validation and consumer acceptance. A wide array of yucca extract is marketed as capsules, beverages and herbal tea. 'Now Foods', markets a supplement rich in saponins for amelioration of joint pains. 'Nature's way' manufactures a nutraceutical from *Y. schidigera* stalk. 'Calivita' sells a powder supplement under the brand name 'Pure yucca' to bolster metabolism and detoxification of gut. 'Walmark' trades *Y. elata* extract claiming its potency to boost metabolism, retard thrombosis and atherosclerosis risks, and ameliorate skin problems. 'Gluten-free remedies' sells root extract of *Y. schidigera* to support normal flora of intestine and improve immune status. 'Health Aid' markets *Y. filamentosa* root. High-grade *Y. schidigera* root extract has a considerable number of uses owing to the prolific plant saponins. Pharma giant 'Swanson' markets yucca capsules for amelioration of joint pain.

### 1 Phytochemicals and Mechanisms of Action

Virtually every part of yucca possesses bioactive compounds *viz.* leaves, shoots, rhizomes, seed pods, flowers and bark. Several phytochemicals of therapeutic importance have been reported in recent times. Saponins are vital components of yucca with broad-spectrum biological properties. These

*To whom correspondence should be addressed. E-mail: seemabiotech83@gmail.com

**Figure 1.** Figures of some important yucca species steroidal metabolites found in the shoots and rhizomes play key roles in food, cosmetics and pharmaceuticals. Saponins are surface-active sterol or triterpene glycosides with both polar and non-polar moieties. Their antibiotic, haemolytic and hypocholesterolaemic activity and ability to form stable foams in aqueous solution are well documented. The amphipathic nature renders them with surfactant property employable for enhancing penetration of proteins through cell membranes. The ability to reduce surface tensions of substances enables them to regulate blood clots, reduce thrombosis and eliminate heart attack risks. Further, they act as adjuvants for vaccines. Some saponins have been shown to enhance cytotoxicity of drugs against cancer. Steroidal saponins in yucca plant parts are precursors to cortisone, thus believed and to some extent, observed to provide relief from arthritis and rheumatism pain, as in case of *Y. schidigera* and *Y. valida*. Also, many polyphenolics including resveratrol and a number of other stilbenes have been reported. The phenolic compounds, yuccaols A, B, C, D and E have been isolated from yucca bark.[1] Acid-hydrolysed fractions of yucca contain both furostanol and spirostanol aglycones. From the seeds of *Y. glauca* Nutt., sapogenins as sarsasapogenin, markogenin, tigogenin, neo-tigogenin, neo-gitogenin, hecogenin and gloriogenin have been isolated and identified. Ethanolic extract of *Y. aloifolia* yielded a new spirostanol glycoside.[2] The nuclear transcription factor NFκB, stimulates the synthesis of inducible nitric oxide synthase (iNOS), which causes formation of the inflammatory agent nitric oxide. Yuccaols are inhibitors of the NFκB, thus anti-inflammatory by nature.

### 2 Food and Pharmaceutical Prospects

A substantial number of published reports exist to lend credence to the functional food and pharmaceutical potentials of various yucca species. The biological roles of most-studied yucca species have been presented in Table 1.

#### 2.1 Food Additives

Yucca extracts have acquired GRAS (Generally Recognized As Safe) status, thus approved for incorporation in foods. A saponin fraction was prepared from yucca for food use.[3] The influence of water extract of *Y. schidigera* powder was evaluated on the foaming potential, structure of candy-foam and its stability during the shelf-life. The steroidal saponin containing extract exerted visible effect on structural attributes *viz.* dry matter content, hardness, density and porosity. The shelf-life was better than that of α-tocopherol, the freshness retained in a two month period observation.[4] The foaming potential of the extract in beverages and soft drinks has been reviewed.[5]

Mechanically sound and moisture-resistant films are sought in food industries for efficient packaging. The possibility of recruiting *Y. schidigera*-derived surfactant in manufacturing gelatine-based film was assessed.[6] On extrusion, it demonstrated good solubility to produce homogeneous films. Also, it showed better water barrier properties due to the characteristic hydrophobicity of the steroidal moiety of the saponin. However, the desired plasticizing effect was not obtained leading to brittleness which requires further investigation or external plasticizers.

#### 2.2 Antioxidant

It was studied that the methanol extract of the bark of *Y. periculosa* F. Baker, the antioxidants stilbenes and resveratrols.[7] It was observed that resveratrol and the phenolics yuccaols A and C from the bark of *Y. schidigera* inhibits free radical generation in blood platelets and protect against cardiovascular diseases.[8] The cisplastin-induced oxidative stress-counteracting effect of two phenolic compounds, stilbene and resveratrol sourced from *Y. schidigera* were assessed on human blood platelets and peripheral blood lymphocytes.[9] Considerable reduction in oxidation of thiol groups of proteins was reported. The antioxidant and anti-inflammatory properties of yuccaols (A, B, and C) isolated from *Y. schidigera* bark were validated further.[10] LC/ESIMS (liquid chromatography coupled to electrospray mass spectrometry) qualitative and an LC/ESIMS/MS (liquid chromatography coupled to tandem electrospray mass spectrometry) quantitative proved to be the ideal strategy for separation and determination of yuccaols and gloriosaols. TEAC assay and ESIMS, NMR data confirmed that gloriosaols A–E extracted from *Y. gloriosa* has antioxidant activity.[11,12] *Y. schidigera* and *Y. gloriosa* roots and barks contained these bioactive phenolics.[13] The protective effects of *Y. schidigera* was assessed against oxidative damage induced by acute nitrite intoxication in murine models.[14] The treatment with the extract was found to decrease methemoglobin, blood and tissue malondialdehyde, and tissue nitric oxide concentrations. Further, it increased the glutathione in blood and various tissues. Contrary to the control group, only moderate or mild haemorrhage and hyperaemia (abnormal amount of blood in the capillaries) were seen in kidneys of yucca-fed group. It was inferred that the extract can be used to lessen nitrite-induced oxidative damage and allied complications. The effect of dietary supplementation of *Y. schidigera* on lipid peroxidation was determined in arsenic-exposed mice. The group administered with different

**Table 1. The functional compounds and biological roles of some medicinal Yucca species**

| Plants | Bioactive compound | Therapeutic value | Reference |
|---|---|---|---|
| *Yucca schidigera* | Saponin; Yuccaol C; Yuccaone A | Animal health; Sugar-candy foam<br>Anti-inflammatory; Antiproliferation;<br>Hypocholesterolemic; Antidiabetic | Holtshausen et al. 2009; Sucharzewska et al. 2003<br>Marzocco et al. 2004; Balestrieri et al. 2006<br>Kimet et al. 2003; Fidan and Dundar 2008 |
| *Yucca gloriosa* | Gloriosaols A and B | Antioxidant | Montoro et al. 2010; Montoro et al. 2008<br>Bassrello et al. 2007a; Bassrello et al. 2007b |
| *Yucca periculosa* | Resveratrol, Stilbene | Antioxidant | Torres et al. 2003 |
| *Yucca schottii* | Yuccagenin | Antiinflammatory | Backer et al. 2006 |
| *Yucca elephantipes* | Spirostanol glycosides | Antifungal | Zhang et al. 2008 |
| *Yucca glauca* | | Antitumor | Mouli et al. (2009) |
| *Yucca smalliana* | | Antimicrobial | Jin et al. (2007) |

doses of yucca in diet and arsenic in drinking water for a period of 28 days resulted in reversal of the induced-oxidative stress and peroxidation. Also, the activities of antioxidant enzymes increased. Further, protection on focal gliosis (a localized accumulation of glial cells) and hyperaemia in brain, necrosis and degeneration in liver, kidney and heart tissue were observed.[15]

### 2.3 Cytotoxicity

Yuccaols (A, B, and C) obtained from *Y. schidigera* bark were tested on cell proliferation, migration and platelet-activating factor (PAF) biosynthesis. PAF, a potent mediator of inflammation, is known to promote angiogenesis and *in vitro* migration of endothelial cells and Kaposi's sarcoma cells. Yuccaols completely inhibited the VEGF-stimulated PAF biosynthesis and enhanced its degradation. Western blot analysis revealed that yuccaols reduces the VEGF-induced phosphorylation of p38 and p42/44, thus indicating a possible interference with the mechanism underlying the VEGF-stimulated cell proliferation.[10] The antitumor activity of *Y. glauca* against B16 melanoma has been reviewed.[16]

### 2.4 Antiinflammamtory and Antiarthritic

The anti-inflammatory property of *Y. schidigera* has been attributed to the resveratrol and yuccaol C content. It reduced the iNOS protein expression via the transcription factors NFκB in *E. coli* LPS-activated macrophage cell line.[1] It was showed that the saponin-containing fraction of the leaves of *Y. schottii* Engelm. exerts antiinflammatory properties against carrageenin-induced edema in rats.[17]

### 2.5 Antidiabetic

The effects of *Y. schidigera*, *Quillaja saponaria* and a mixture of both plants were examined on streptozotocin-induced diabetic rats. After feeding for three weeks, the blood glucose level was found to be significantly lower. The insulin levels increased and the plasma cholesterol and triglyceride levels significantly decreased. Mononuclear leukocyte DNA damage, plasma malondialdehyde, and plasma protein carbonyl levels were found to be significantly lower.[18]

### 2.6 Hypocholesterolaemic

The effects of a blend of partially-purified *Y. schidigera* and *Quillaja saponaria* extracts on cholesterol levels in the human blood was studied. Taking 0.9 mg of the mixture (6:4, v:v) a day for a month resulted in decrease of total and LDL cholesterol levels in blood plasma of hyper-cholesterolaemic patients.[25]

### 2.7 Antimicrobial

Antiprotozoal role of saponins have been oft-proved. A saponin fraction from the stems of *Y. schidigera* exhibited inhibition against certain food-deteriorating, film-forming and dermatophytic yeasts and fungi.[20] The antigiardial effect of oral intake of *Y. schidigera* powder was investigated by enumeration of gut trophozoites and faecal cysts in infected gerbils and lambs. The butanol extract (6.1 mg) or metronidazole (1 mg) taken once daily for three days reduced the number of trophozoites in the small intestine of infected gerbils. Oral dosing of 50 mg of the extract in eight doses over a period of 3 days reduced trophozoites in the duodenum and jejunum, and eliminated them from the ileum. 10 g powder/day showed fewer cysts shedding. Further purification may exert more significant anti-giardial effects.[21] The gastro-intestinal tract responded to the anti-protozoal activity of saponins. The saponins of *Y. schidigera* proved as effective as the drug metronidazole in killing tropozoites of *Giardia lamblia* in the intestine. The mechanism of action was deduced to be due to binding of saponins with microbial membranes and causing pores in them.[22] It was discovered that the leaf extract of *Y. smalliana* Fern. has also antimicrobial property.[23] The steroidal saponin, yuccalan demonstrated pronounced antifungal activity against *Rhizoctonia solani* and *Fusarium oxysporum*. Two spirostanol glycosides isolated from *Y. elephantipes* showed moderate inhibitory activity against *Candida albicans* and *Cryptococcus neoformans*.[24] An anti-mycotic substance, gloriofucin was prepared that contained spirostanol glycosides, extracted from the dried leaves of *Y. gloriosa*.[25]

## 3 Enhancement of Bioactive Extraction

Extraction of the bioactive constituents by traditional method as Soxhlet serves the purpose; however, for economical efficiency advanced methods prove useful. Ultrasound-assisted extraction yielded better saponin content from ginseng compared to the conventional method. Microwave-assisted extraction using 80% methanol could dramatically reduce the extraction time of ginseng saponins from 12 h using conventional extraction methods to a few seconds.[26] Ultrafiltration and sequential diafiltration can enhance the concentration of saponins of *Y. schidigera* from 9.3% to 17.2%.[25] In an attempt to enhance metabolites sapogenins, *Y. Schidigera* rhizome organ culture was conducted.[27] However, the quantification by HPLC did not show any significant improvement in addition of growth regulators.

## 4 Discussion

Ample literature exists on the therapeutic potency of steroidal saponins from varied plant sources. Steroidal saponins isolated from the roots of *Asparagus acutifolius* L. have showed antifungal activity against various Candida pathogens.[28] The steroidal saponins from *Agave utahensis* have been reported to show strong cytotoxic activity against HL-60 human promyelocytic leukemia cells.[29] Total steroidal saponins extracted from the rhizomes of *Dioscorea zingiberensis* exert healing effect on cardiovascular disease. It shows anti-thrombotic activity by prolonging the bleeding time and clotting time in a dose-dependent manner in mice.[30] Steroidal saponins isolated from the rhizomes of *Paris polyphylla* var. Yunnanensis demonstrated superior cytotoxic effects than cisplatin on human nasopharyngeal carcinoma epithelial cells. The saponins with diosgenin and tetrasaccharide moieties exerted the robust inhibitory effect mediated by apoptosis and cell cycle arrest.[31] The saponins from various yucca species can also be explored for wide spectrum therapeutic potential. There exist sparse findings on the antitumor potential of *Y. glauca* Nutt., herpes simplex virus type 1 inhibition of yucca leaf protein, antimutagenicity of resveratrol from *Y. schidigera* against *Salmonella typhimurium*, anti-yeast action of spirostanol saponins from *Y. schidigera*. Research undertakings in these promising aspects must be revived.

Going by the yucca-based dietary supplements being commercialized, health attributes of this genus are evident. The emerging clinical evidences of the therapeutic role calls for more research attention. Out of the 40–50 species only a few have been medicinally validated. The *Y. brevifolia* species, found abundantly in the Mojave National Preserve has been scarcely explored and can be an interesting subject for pharmaceutical discovery. Steroidal saponins have wide structural diversity and significant biological activities. Yucca saponins deserve more investigations in the interest of broader medicinal findings. Traditional knowledge of Native Americans could be consulted for research initiatives. Pharmacognosy will certainly be richer by contribution from this genus. However, the claims must be scientifically verified with more emphasis on human data.

**Open Access** This article is distributed under the terms of the Creative Commons Attribution License which permits any use, distribution, and reproduction in any medium, provided the original author(s) and source are credited.
